# Presidential Address of Dr. A. V. Elliott, Florence, Italy

**Published:** 1896-10

**Authors:** 


### PRESIDENTIAL ADDRESS OF DR. A. V. ELLIOTT, FLORENCE, ITALY.[1]

My Brethren,—On taking the presidential chair of this, the Florentine Dental Society of Italy, for the new term just commencing, allow me to express to you all my heartfelt thanks for the honor thus conferred upon me. I look upon this act on your part as a singular manifestation of good will, the more so—although I say it to my shame—that after all these years I have been among you I do not speak, as I should, your most pleasing and musical language. But, thanks to the kindness and courtesy of your worthy founder, I will be enabled to convey through him the expression of my gratitude and good-will towards you, and at the

---

[1] Delivered before the Florentine Dental Society, Italy, February 26, 1896.

same time say a few words in regard to our enlightened and useful profession.

Not only are we ourselves an educated body of men in our specialty, but we are at the same time educators. A great responsibility rests with us in this respect. In regard to their teeth, the people are what the dentists make them; and to a great extent the compliment is returned in the fact that the dentists are also to a great extent what the people make them. A class of people who are satisfied with, and even prefer, slovenly and cheap work to the best efforts of a dentist will, as a general thing, get slovenly and poor work as a result. Good work is not encouraged or appreciated by them, questions of pain, time, and pay being of more consequence than thorough and permanent operations. It follows, therefore, that, under the all-pervading law of least resistance, the general tone of the profession among such people is lowered; so, in all seriousness, let me say that it is our *special* duty to society and to ourselves to do all we can to educate and enlighten those within our reach as regards the advantages to them of securing the best possible efforts from us professionally, and thus reap for ourselves such golden rewards as a higher standard of work would naturally command.

It is not because I am an American that I state the fact that the American people, as a people, take better care of their teeth than do any other people, but because it is a fact to which any one present who has had occasion to work in the mouths of Americans can bear testimony. The truth is that the American people, as a general thing, demand a higher excellence in the care of their teeth than any other; and they, by so doing, encourage and support the best efforts of the members of our profession, who in their turn, being thus encouraged and supported, do their best, by their work, teaching, and advice, to uphold the high standard thus demanded. In this the American dentist is aided by the fact that there is no social discrimination against him, for a dentist with us is as good as any one else, provided, of course, that he as a man *is* as good as any one else, and in consequence his influence has the importance it deserves.

It is not for me, an American, to claim any especial superiority for so-called "American dentistry." As a matter of fact, there is no such discriminating title as "American dentist" in America. All are simply dentists, be they good, bad, or indifferent. Only this, a good conscientious dentist is sure, sooner or later, to be found out and his best efforts stimulated by the higher prices he can obtain for his work. There need be no limit for his ambition. It is all within himself. There is always "room at the top." Nor is it necessary, in my opinion, that a man should be born in America to be a good dentist, for, as a matter of fact, some of the best dentists in America, receiving the highest fees, are of foreign birth. In America, as in Italy, there is no prejudice against the foreigner. It is the *man*, not the country where he was born, which gives him success or failure. We look upon it as an advantage to have a mixture of races at our meetings, bringing with them the good things culled from the books and magazines of their own native lands. And in this connection I am glad to see that here, in old Italy, you, too, appreciate such advantages.

